# Supplementary material for: Osteocyte transcriptome mapping identifies a molecular landscape controlling skeletal homeostasis and susceptibility to skeletal disease
Source: Nat Commun. 2021 May 5;12:2444. doi: 10.1038/s41467-021-22517-1 (PMC8100170; doi:10.1038/s41467-021-22517-1)
Supplement: Supplementary file 1 — Supplementary Information [file 41467_2021_22517_MOESM1_ESM.pdf]

Supplementary Figure 1

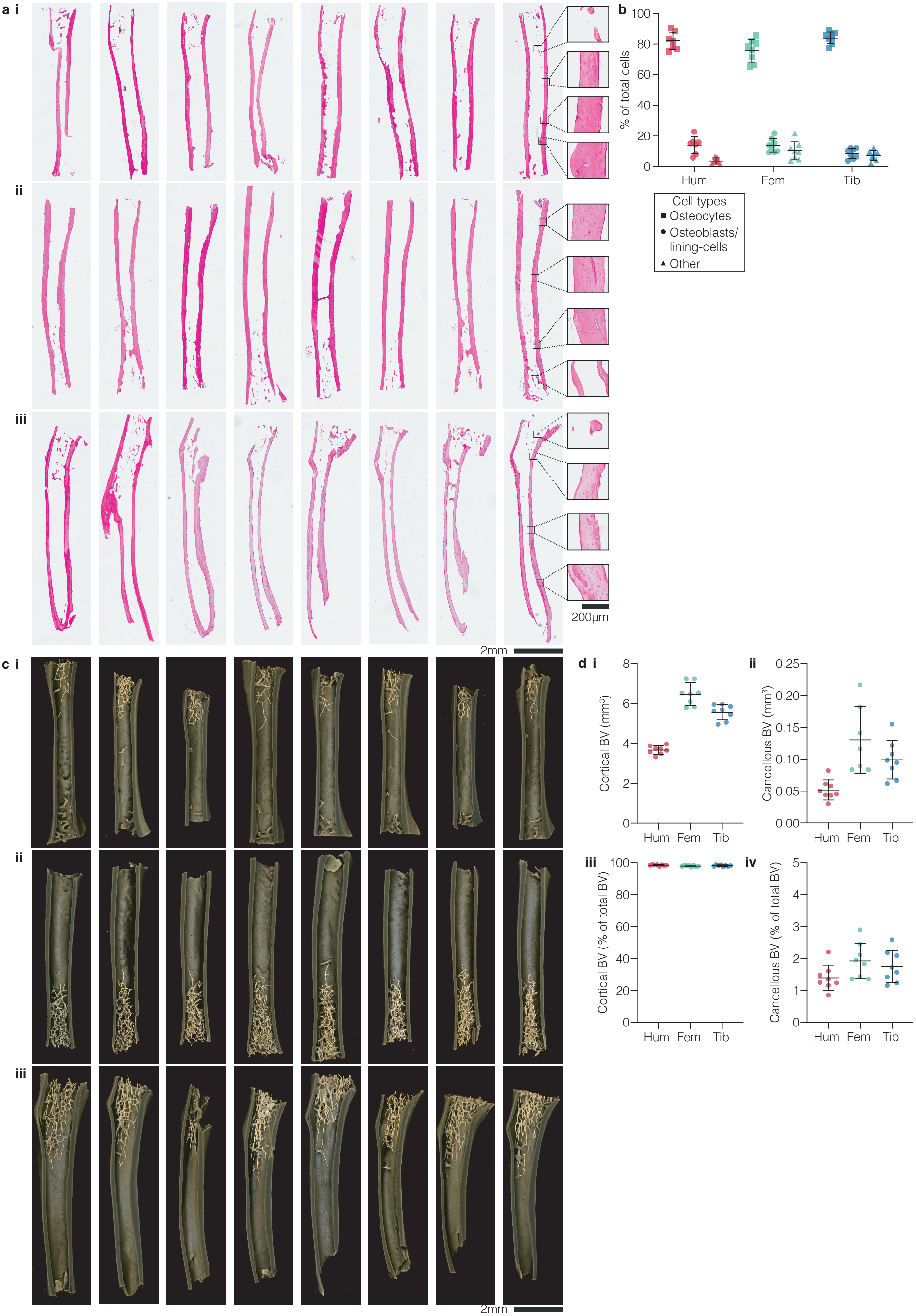

Supplementary Fig. 1: Osteocyte enrichment in processed bone samples.

a, Histology images of bone samples from the humeri (Hum) (i), femora (Fem) (ii) and tibiae (Tib) (iii). Example high-magnification images show the effective removal of marrow and endosteal cells and enrichment for osteocytes. **b,** Quantitative analysis of bone-cell types present in osteocyte-enriched samples. Other denotes all cell types that could not be defined as osteoblasts, lining cells or osteocytes. Empty lacunae were excluded. Individual data points are shown. Each point represents data from a single bone from one mouse. Error bars show mean +/- SD.**c,** Micro-CT images of osteocyte-enriched bone samples from the humeri **(i)**, femora **(ii)** and tibiae **(iii)** following processing. Samples correspond to those in panel **(a)** above. Samples were collected from the contralateral limb and processed at the same time as those used for transcriptome sequencing. **d,** The cortical **(i)** and cancellous **(ii)** bone volume (BV) in processed bone samples from each skeletal site, and the relative proportion of total BV (as a percentage) of each bone type **(iii-iv)**. For **a**-**d**, n=8 biologically independent mice per bone type. Data are presented as mean values +/- SD.

Supplementary Figure 2

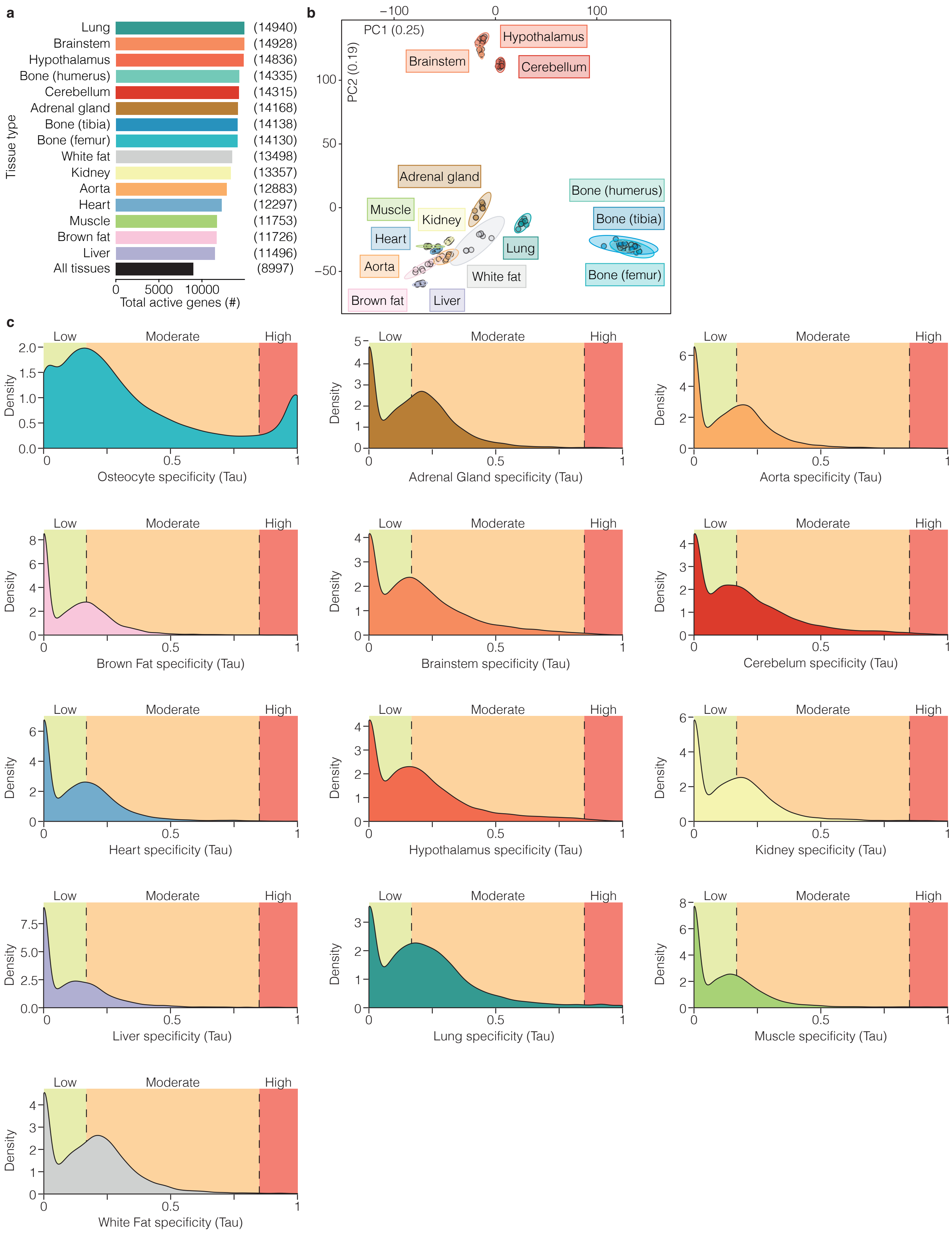

Supplementary Fig. 2: The osteocyte transcriptome is distinct from transcriptomes expressed by other organs and tissues

a, The number of genes actively expressed in osteocytes from the humerus, tibia and femur, and 12 other organs and tissues[28]. The black bar indicates the number of genes common to all tissues. **b,** Principal component (PC) analysis of active gene expression in osteocytes isolated from the humerus, tibia and femur, and 12 other organs and tissues. Dots represent individual biological replicates (n=8 mice per sample type) and ellipses represent 95% confidence intervals for each sample type. Coloured labels correspond with sample type. The percentage of total variance explained by individual PCs is shown. **c,** The distribution of active gene expression specificity[29] (Tau) in osteocytes and 12 non-skeletal organs and tissues, calculated for each gene in the osteocyte transcriptome. Genes with Tau < 0.15 have low expression specificity in a given tissue (green), 0.15 ≤ Tau ≤ 0.85 have moderate specificity (orange), while Tau > 0.85 have high expression specificity (red).

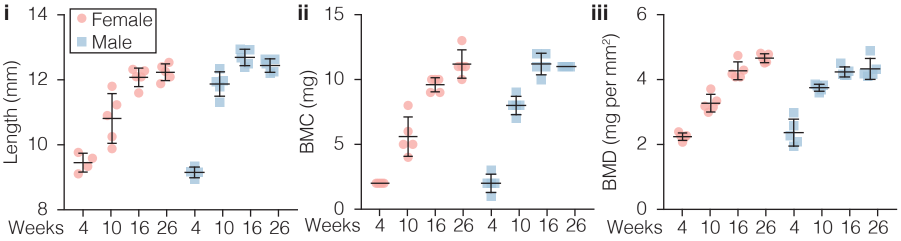

Variation (%)	P value
Age (86.6)	<0.0001*
Sex (2.4)	0.003*
Interaction (3.7)	0.005*

Variation (%)	P value
Age (92.4)	<0.0001*
Sex (1.6)	0.001*
Interaction (2.1)	0.003*

Variation (%)	P value
Age (91.0)	<0.0001*
Sex (0.1)	0.451
Interaction (2.7)	0.009*

Supplementary Fig. 3: Differences in bone structure during skeletal maturation in both sexes

Bone structural parameters, humeri length **(i)**, bone mineral content (BMC) **(ii)** and bone mineral density (BMD) **(iii)** from female (pink) and male (blue) mice at different ages (weeks). Individual samples are denoted by dots and the mean ±SD are shown. Statistical analysis performed by two-way analysis of variance (ANOVA). Percentages represent the variance in each parameter with age, sex and the interaction between age and sex. n=5 mice per age for each sex.

Supplementary Figure 4

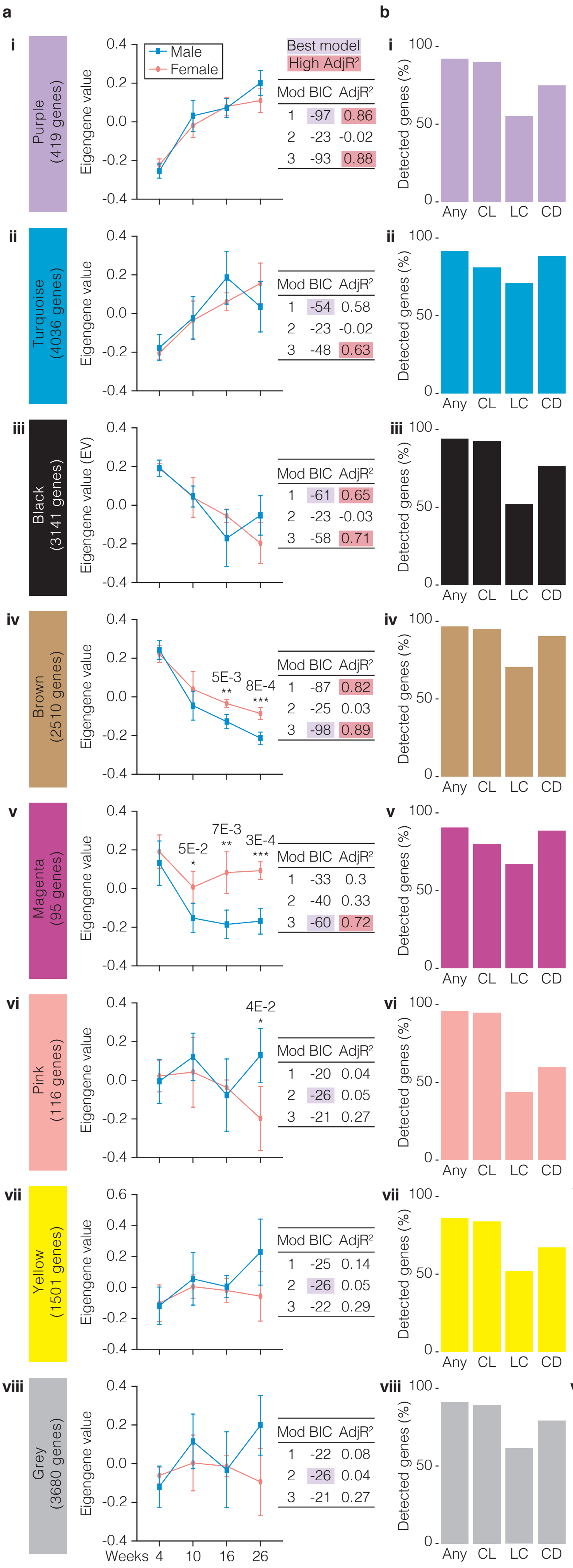

c

Module	Database	Term description	P value
i Purple	GOBP	None significant	
	KEGG	None significant	
	DO	DOID:28 Endocrine system disease	3.2E-02
ii Turquoise	GOBP	GO:0045087 Innate immune response	9.9E-46
		GO:0050778 +ve regulation of immune response	4.5E-32
		GO:0098542 Defense response to other organism	4.6E-29
	KEGG	mmu05322 Systemic lupus erythematosus	9.8E-28
		mmu05332 Graft-versus-host disease	5.9E-09
		mmu03030 DNA replication	1.7E-08
	DO	DOID:74 Hematopoietic system disease	2.7E-10
		DOID:0060056 Hypersensitivity reaction disease	1.6E-09
		DOID:104 Bacterial infectious disease	2.3E-08
iii Black	GOBP	GO:0048598 Embryonic morphogenesis	2.4E-13
		GO:0007224 Smoothened signaling pathway	2.7E-13
		GO:0048729 Tissue morphogenesis	5.6E-12
	KEGG	mmu04390 Hippo signaling pathway	2.1E-09
		mmu04360 Axon guidance	1.4E-06
		mmu04550 Stem cell pluripotency*	1.1E-04
	DO	None significant	
iv Brown	GOBP	GO:0048193 Golgi vesicle transport	6.8E-11
		GO:0006888 ER to Golgi vesicle-mediated transport	1.9E-09
		GO:0006487 Protein N-linked glycosylation	9.7E-06
	KEGG	mmu04141 Protein processing in ER*	6.9E-13
		mmu00510 N-Glycan biosynthesis	1.8E-05
		mmu03060 Protein export	2.7E-04
	DO	None significant	
v Magenta	GOBP	GO:0015991 ATP hydrolysis coupled proton transport	2.1E-14
		GO:0099131 ATP hydrolysis coupled ion transport*	2.1E-14
		GO:0099132 ATP hydrolysis coupled cation transport*	2.1E-14
	KEGG	mmu04142 Lysosome	2.2E-17
		mmu05323 Rheumatoid arthritis	9.0E-14
		mmu04721 Synaptic vesicle cycle	5.4E-13
	DO	DOID:13533 Osteopetrosis	6.2E-07
		DOID:4254 Osteosclerosis	4.7E-06
		DOID:3211 Lysosomal storage disease	1.2E-03
vi Pink	GOBP	GO:0071559 Response toTGF-beta*	1.6E-02
		GO:0015914 Phospholipid transport	3.3E-02
	KEGG	mmu04550 Stem cell pluripotency*	2.9E-04
		mmu05205 Proteoglycans in cancer	2.3E-02
	DO	None significant	
vii Yellow	GOBP	GO:0006091 Generation of metabolites and energy*	3.1E-49
		GO:0015980 Energy derivation by oxidation*	6.0E-48
		GO:0045333 Cellular respiration	1.6E-38
	KEGG	mmu05012 Parkinson disease	4.4E-38
		mmu00190 Oxidative phosphorylation	2.7E-37
		mmu04714 Thermogenesis	5.8E-36
	DO	DOID:423 Myopathy	2.4E-16
		DOID:66 Muscle tissue disease	2.4E-16
		DOID:0080000 Muscular disease	8.2E-16
viii Grey	GOBP	GO:0034660 ncRNA metabolic process	1.4E-21
		GO:0034470 ncRNA processing	3.9E-21
		GO:0022613 Ribonucleoprotein biogenesis*	3.8E-17
	KEGG	mmu03010 Ribosome	2.4E-13
		mmu03008 Ribosome biogenesis in eukaryotes	1.6E-04
		mmu03018 RNA degradation	3.7E-04
	DO	None significant	

Supplementary Fig. 4: Gene clusters co-regulated during skeletal maturation

a, Pattern of gene expression change for each cluster (summarised by eigengenes) during postnatal skeletal maturation in male (blue) and female (red) mice **(i-viii)** (n=5 mice each age/sex). Significant separation of eigengene values between sexes at each age is indicated (* $p<0.05$, ** $p<0.01$, *** $p<0.001$). P-values calculate by multiple t-tests followed by Bonferroni-Dunn's multiple comparison adjustment. Data are presented as mean values +/- SD. Three separate linear models (Mod) fitting eigengene variance (EV) for each colour cluster (sex-only $EV_{cluster}$~Sex, age-only $EV_{cluster}$~Age, full model $EV_{cluster}$~Age + Sex + Age*Sex) to establish association between cluster expression age/sex are shown. The Bayesian Information Criterion (BIC) was used to select the best model fit and adjusted-R^2 ($AdjR^2$) used to estimate model strength. The optimum model (lowest value) selected by BIC is highlighted in purple. Linear models with a high $AdjR^2$ (>0.6), indicating a strong association with eigengene expression, are highlighted in red. **b,** The percentage of expressed genes in each cluster that were also expressed in the osteocytic IDGSW3 cell-line (CL), in laser capture micro-dissected osteocytes (LC) and collagenase-digested bone samples (CD), or ANY of these orthogonal datasets. c, The top three most significantly enriched gene ontology biological processes (GOBP), KEGG pathways and disease ontology (DO) terms in each cluster **(i-viii)** with p-values (Bonferroni adjusted) calculated by hypergeometric test.

Supplementary Figure 5

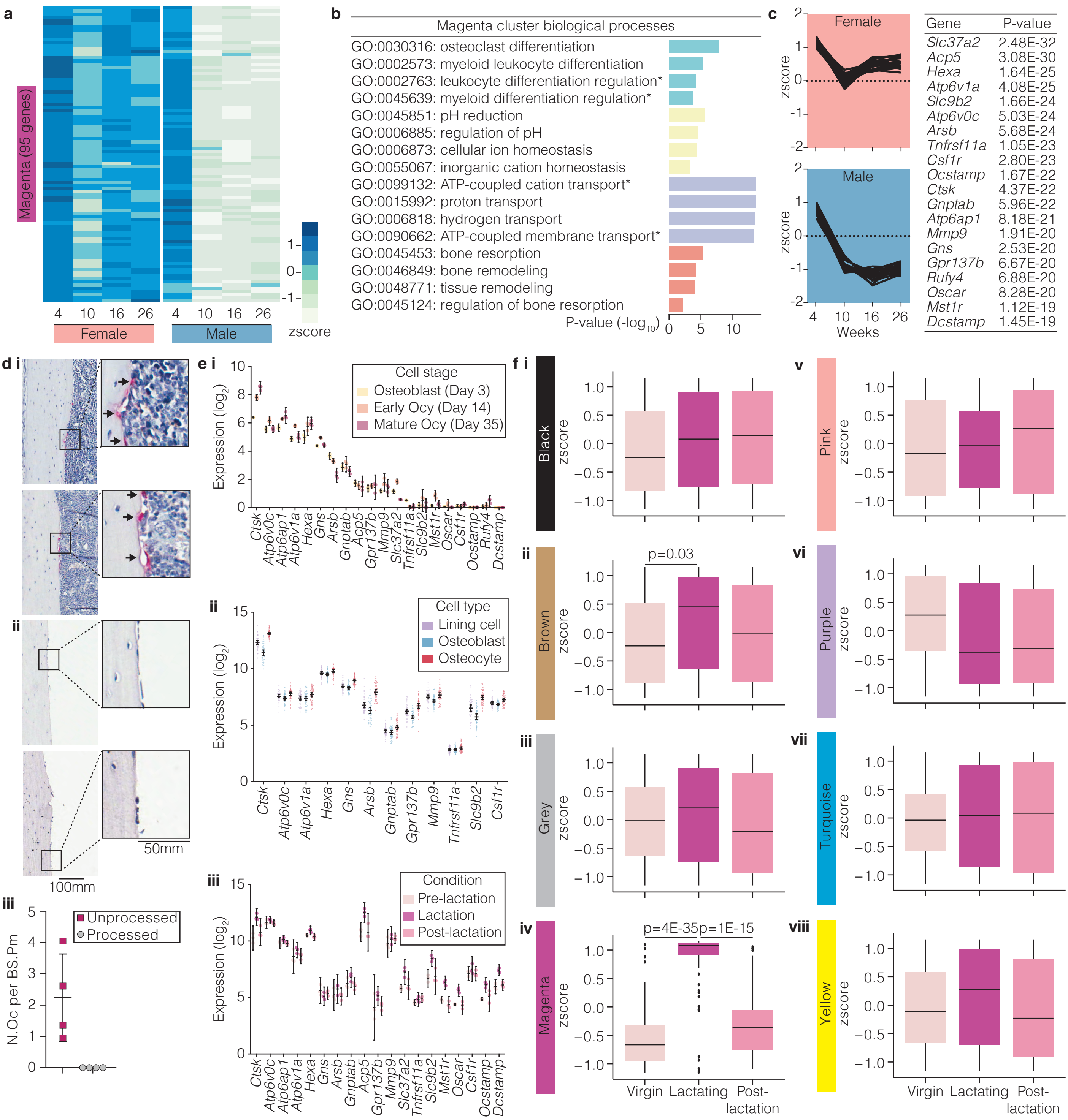

Supplementary Fig. 5: Magenta cluster genes associated with perilacunar-remodeling

a, Heatmap showing Magenta cluster gene expression at different ages (weeks) in male and female mice. Values reflect mean of scaled gene expression values at each time point. **b,** Biological processes significantly overrepresented among Magenta cluster genes grouped by semantic similarity (4 groups denoted by colours, *truncated description). P-values (Bonferroni adjusted) were calculated by hypergeometric test. **c,** Top 20 Magenta cluster genes associated with skeletal maturation in male and female mice. P-values (1-sided, unadjusted, calculated by student's t-test) represent the significance of the bi-weight midcorrelation of each gene with the Magenta eigengene **d,** TRAP-stained histological sections of whole-bone (unprocessed) (i) and osteocyte-enriched (processed) (ii) bone samples. Osteoclasts are indicated by arrows. The number of TRAP-positive osteoclasts on the bone surface (iii). N.Oc per BS.Pm = number of osteoclasts per bone perimeter (mm). Data are presented as mean values +/- SD (n=4 biologically independent mice from a single experiment). **e,** Expression of the top 20 magenta genes in the osteocytic IDG-SW3 cells from 3 independent experiments (n=3) (i), laser capture micro-dissected bone cells (n=40 biologically independent mice from a single experiment) (ii) and collagenase digested osteocytes (n=3 biologically independent mice from a single experiment) (iii).). Data are presented as mean values +/- 95% confidence interval. **f,** Expression of genes associated with each cluster in osteocytes from virgin mice, lactating mice and mice post-lactation (Post-lac)[15]. Tukey box-plots show a summary of median cluster gene expression values in each condition calculated across n=3 biological replicates from a single experiment. Boxes indicate median and interquartile range (IQR) of scaled, normalized gene expression values, whiskers denote values ±1.5*IQR and outlier values beyond this range are shown as individual points. P-values (2-tailed, Benjamini and Hochberg adjusted) were calculated by competitive gene set test accounting for inter-gene correlation (CAMERA)[105].

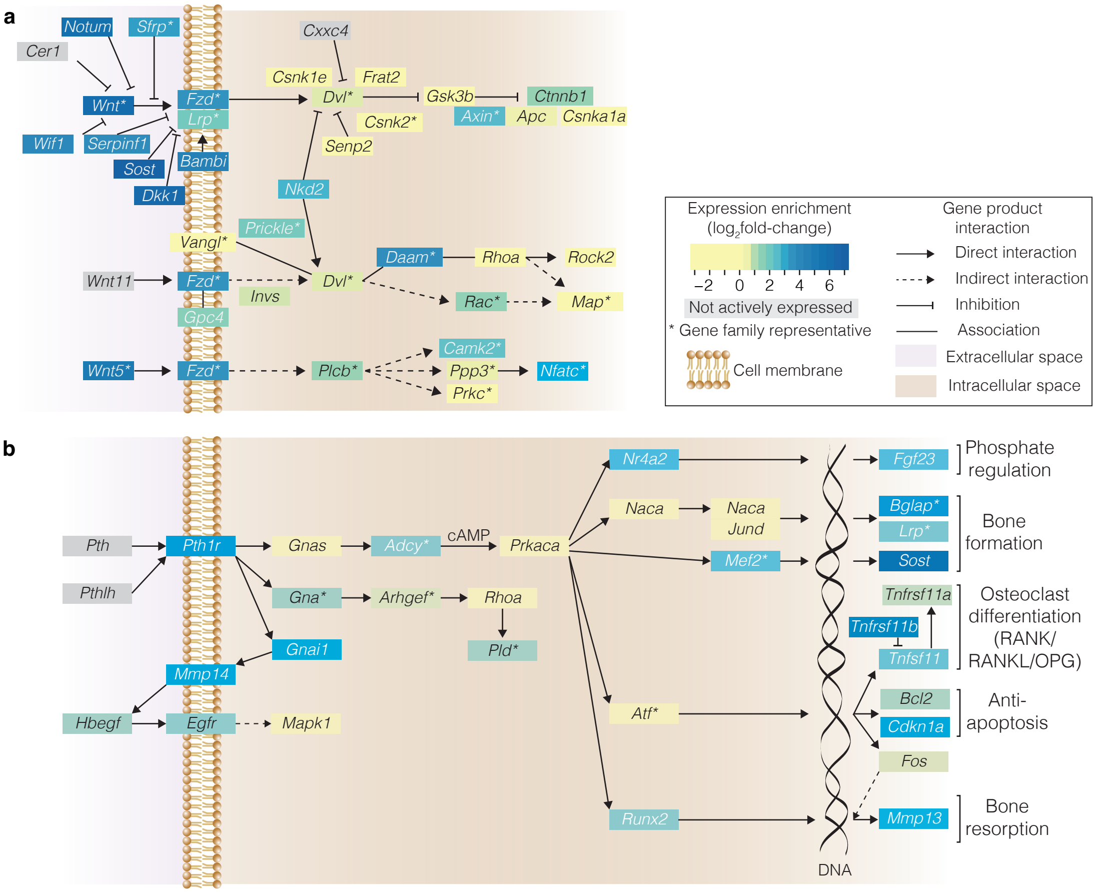

Supplementary Fig. 6: Signaling pathways with established roles in bone are enriched for expression in osteocytes

a, Wnt-signaling and **b,** PTH-signaling pathway genes actively expressed in osteocytes. Level of expression enrichment in osteocytes increases from yellow to blue (log_2-scale). Grey represents genes not actively expressed. Lines and arrows indicate associations, inhibition, and direct or indirect interactions. * denotes most enriched gene where more than one molecule from a family of related genes can function at the same point in the pathway.

Supplementary Figure 7

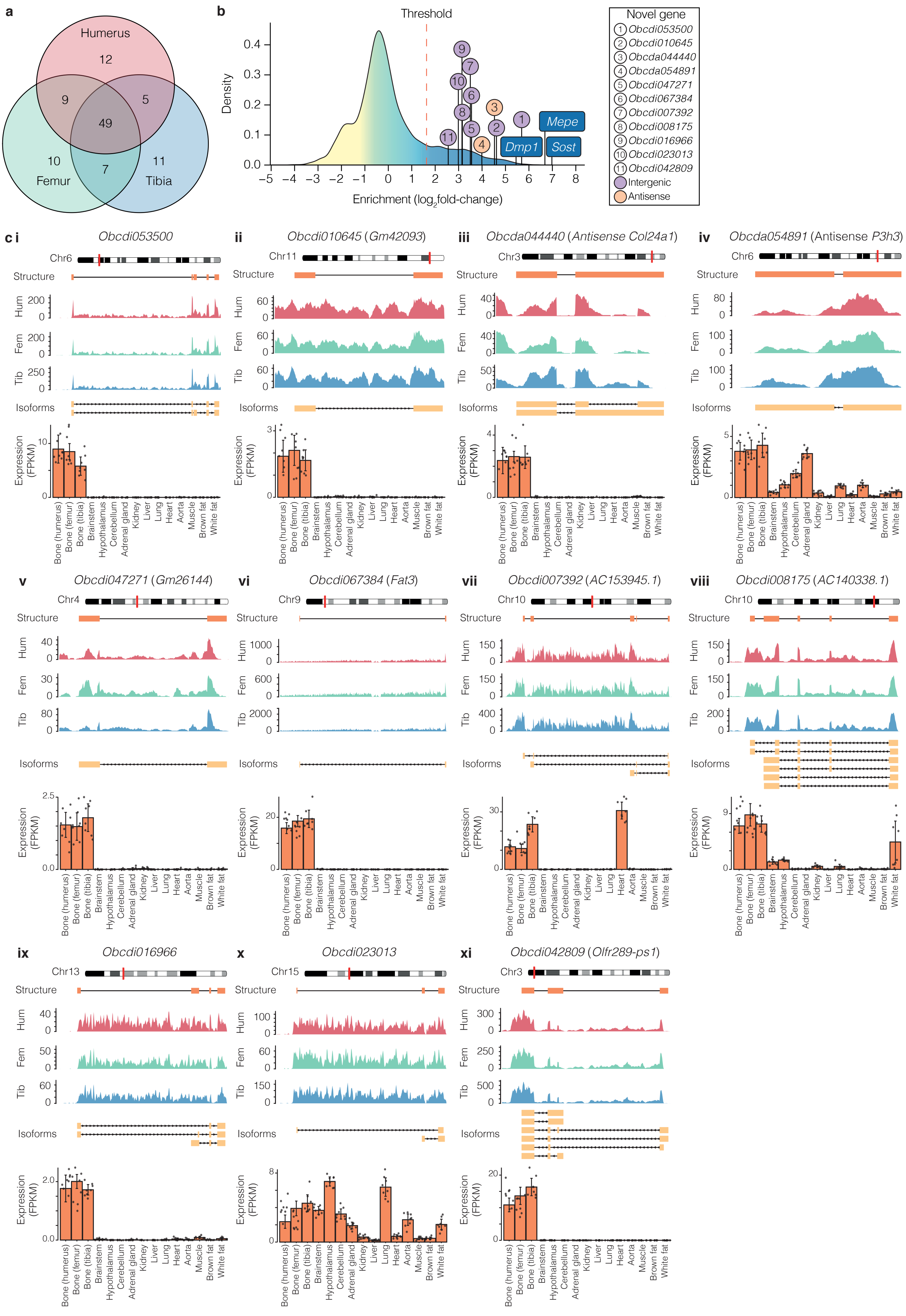

Supplementary Fig. 7: Novel genes identified in the osteocyte transcriptome signature

a, Venn diagram showing the number of novel genes actively expressed in osteocytes from different bone types. **b,** Expression enrichment of 11 novel genes identified in the osteocyte transcriptome signature. Intergenic genes are denoted in purple and antisense genes in orange. Established osteocyte genes are labeled with gene symbols. **c,** Gene structure diagrams of novel genes in the osteocyte transcriptome signature **(i-xi)**. Chromosome and location on each chromosome (red line), pooled read data alignment for humeri (Hum), femora (Fem) and tibiae (Tib) and individual predicted isoforms are shown. Histograms show normalized expression of each gene in osteocytes from three bones relative to 12 organs and tissues[28] (n=8 biologically independent mice per samples type). FPKM = Fragments per kilobase per million mapped reads. Data are presented as mean ± SD.

a

KEGG Term Description	P-value
mmu04360 Axon guidance	7.77E-17
mmu04151 PI3K-Akt signaling pathway	1.41E-13
mmu04512 ECM-receptor interaction	7.31E-10
mmu04510 Focal adhesion	4.03E-08
mmu04390 Hippo signaling pathway	7.33E-08
mmu04974 Protein digestion and absorption	1.60E-07
mmu05165 Human papillomavirus infection	1.70E-07
mmu04015 Rap1 signaling pathway	5.93E-07
mmu04310 Wnt signaling pathway	2.56E-06
mmu04350 TGF-beta signaling pathway	3.50E-05
mmu04014 Ras signaling pathway	2.31E-04
mmu05217 Basal cell carcinoma	2.82E-04
mmu04550 Signaling regulating stem cell pluripotency	6.89E-04
mmu05200 Pathways in cancer	1.27E-03
mmu04392 Hippo signaling pathway - multiple species	1.55E-03
mmu05224 Breast cancer	3.94E-03

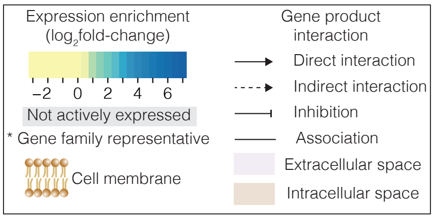

b

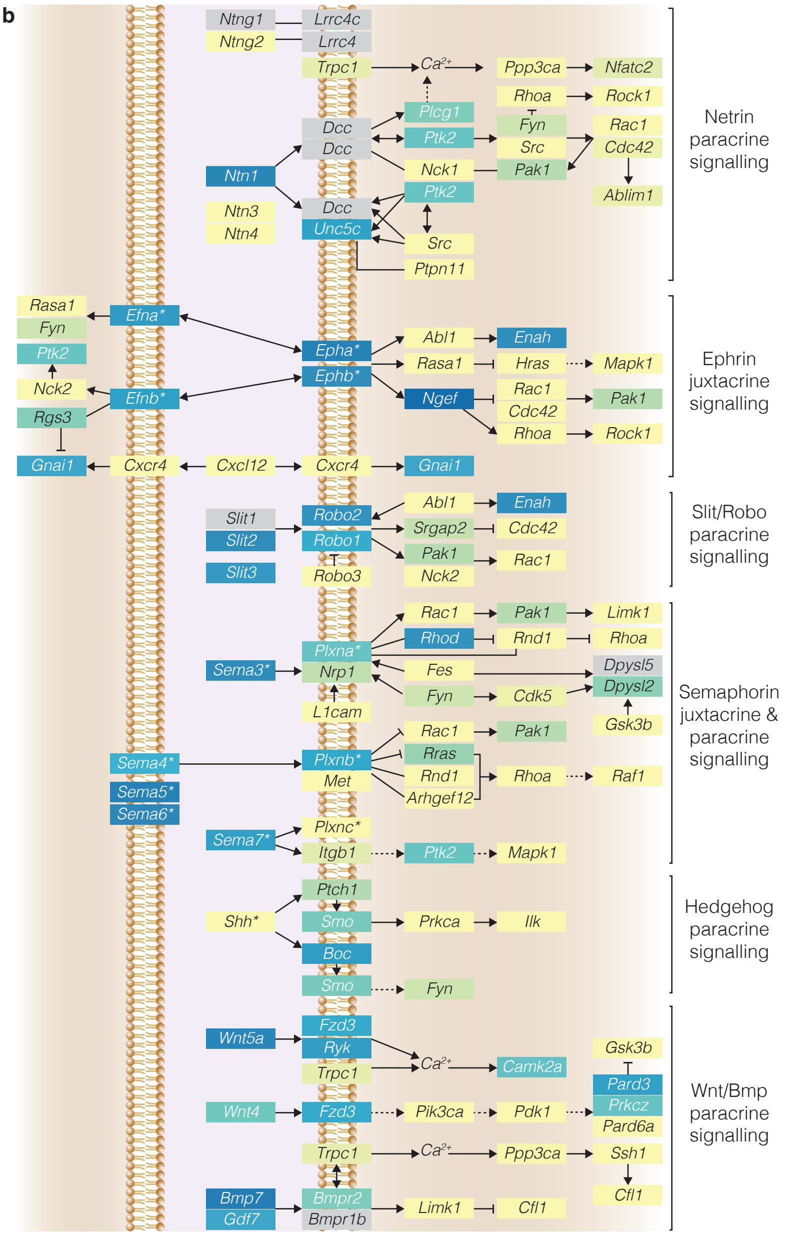

Supplementary Fig. 8: Axon guidance pathway genes are enriched in the osteocyte transcriptome signature

a, Top ranked KEGG pathways significantly enriched among osteocyte transcriptome signature genes and ranked by p-value (Bonferroni-corrected) calculated by hypergeometric test. **b,** Axon guidance pathway genes actively expressed in osteocytes. Level of expression enrichment in osteocytes increases from yellow to blue ($\log_2$-scale). Grey represents genes not actively expressed. Lines and arrows indicate associations, inhibition, and direct or indirect interactions. * denotes most enriched gene where more than one molecule from a family of related genes can function at the same point in the pathway.

Supplementary Figure 9

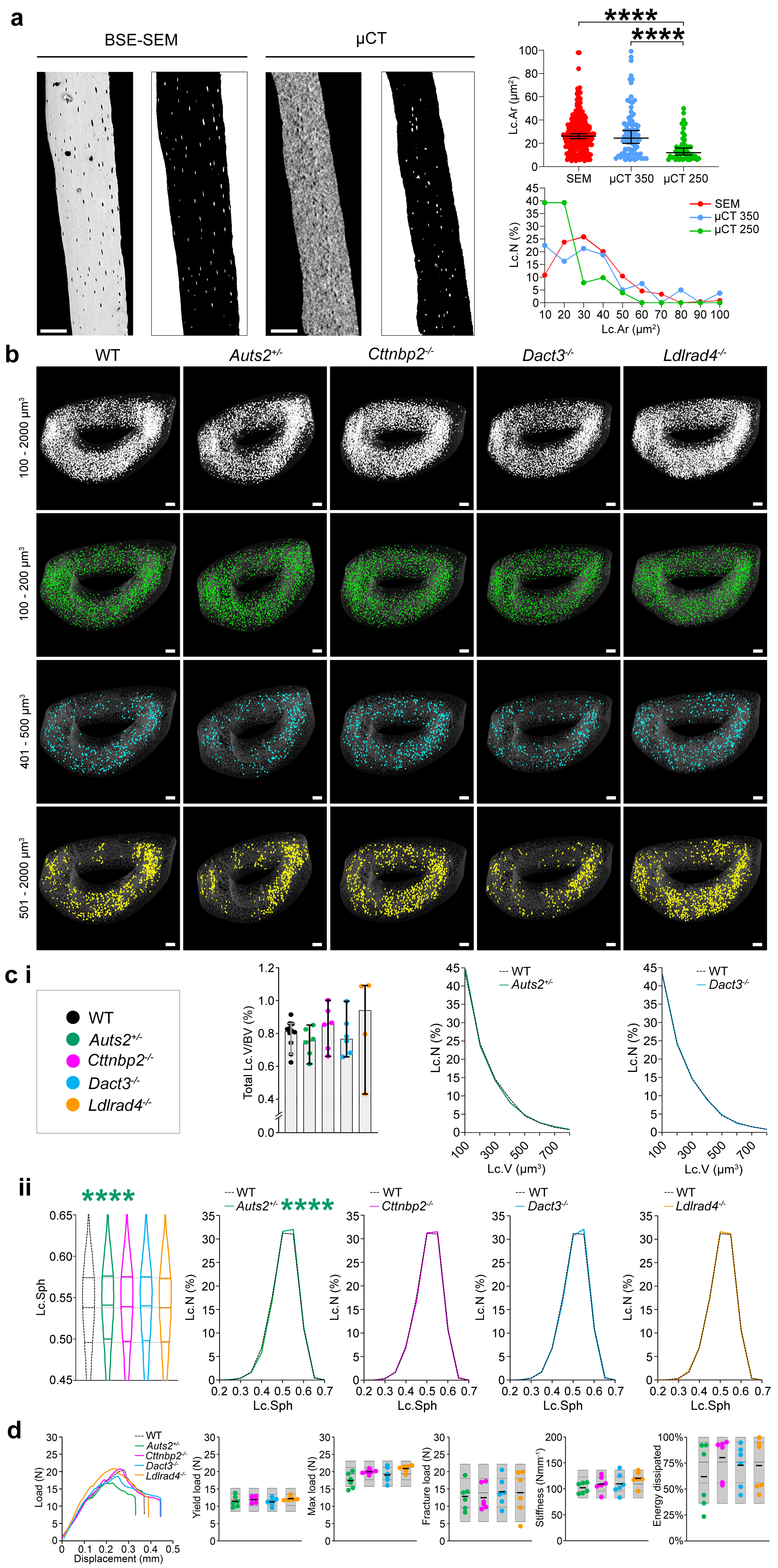

Supplementary Fig. 9: Disruption of the osteocyte network in mice with deletion of osteocyte signature genes

a, Longitudinal back-scattered electron scanning-electron microscopy (BSE-SEM) and micro-CT images of tibia cortical bone from adult female wild type (WT) mice (n=1). Micro-CT image generated at a 1μm voxel resolution using a segmentation threshold of 350. Scale bar= 100μm. The corresponding images to the right show cortical bone in black and osteocyte lacunae in white. Upper graph shows the distribution of individual lacuna area (Lc.Ar), median and 95% confidence intervals when determined by BSE-SEM (n= 240 lacunae) and micro-CT using a segmentation threshold of either 350 (equivalent to 852.72 mgHAcm^{-3}) (n=80 lacunae), or 250 (equivalent to 552.5 mgHAcm^{-3}) (n=48 lacunae). Kruskall-Wallis test followed by Dunn's multiple comparison (2 sided) **** P<0.0001. Lower graph shows the relationship between Lc.Ar and lacunar number (Lc.N). **b,** Mid-tibia micro-CT images from adult female WT (n=11), *Auts2*$^{+/-}$ (n=6), *Cttnbp2*$^{-/-}$ (n=6), *Dact3*$^{-/-}$ (n=6) and *Ldlrad4*$^{-/-}$ (n=4) mice showing distribution of osteocyte lacunae with volumes of 100-2000μm^3, 100-200μm^3, 401-500μm^3, and 501-2000μm^3. Scale bar = 100μm. **c, (i)** Graphs show total osteocyte lacuna volume per bone volume (Lc.V/BV) in adult female WT, *Auts2*$^{+/-}$, *Cttnbp2*$^{-/-}$, *Dact3*$^{-/-}$ and *Ldlrad4*$^{-/-}$ mice (median and 95% confidence intervals) and the distribution of Lc.V in *Auts2*$^{+/-}$ and *Dact3*$^{-/-}$ mice compared to WT. **(ii)** Violin plots and relative frequency graphs show distribution of osteocyte lacuna sphericity (Lc.Sph) in the four knockout mouse lines compared to WT. Kruskall-Wallis test followed by Dunn's multiple comparison (2 sided) **** P<0.0001. **d,** Load displacement curves from femur 3-point bend testing of adult female WT, *Auts2*$^{+/-}$, *Cttnbp2*$^{-/-}$, *Dact3*$^{-/-}$ and *Ldlrad4*$^{-/-}$ mice. Dot plots show yield load, maximum and fracture loads, stiffness and energy dissipated prior to fracture. For each variable the mean (solid centre lines), ±1.0 SD (dotted lines) and ±2.0 SD (grey boxes) for WT mice (n=320) are shown. Mean values for each parameter in *Auts2*$^{+/-}$, *Cttnbp2*$^{-/-}$, *Dact3*$^{-/-}$ and *Ldlrad4*$^{-/-}$ lines are shown as a thick black line and individual data points as green, purple, blue and orange dots respectively (n=6 animals per genotype).

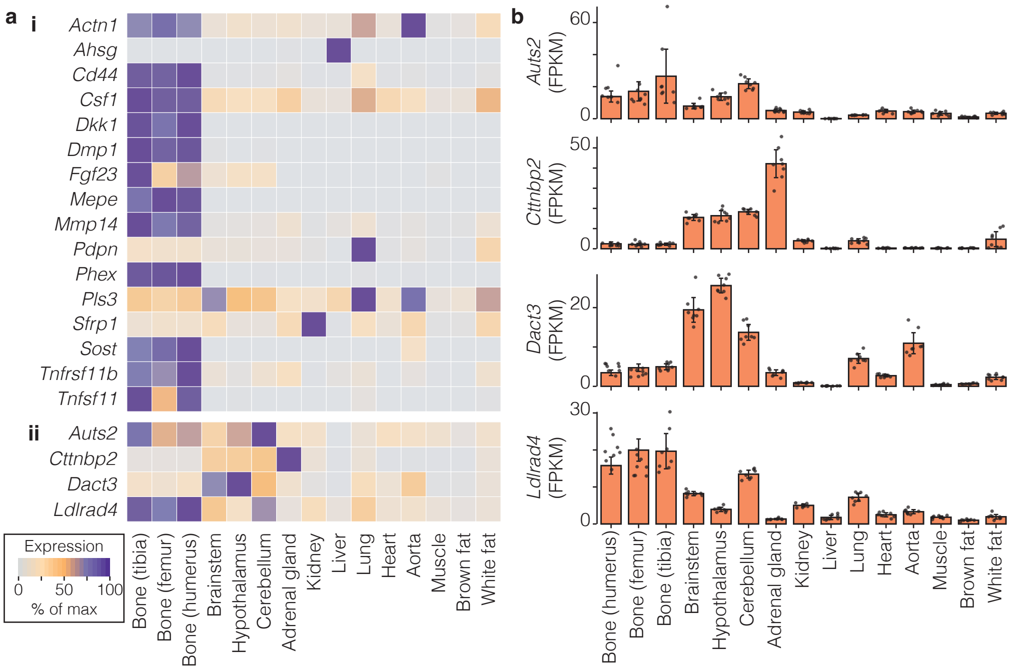

Supplementary Fig. 10: Expression of osteocyte signature genes associated with significant skeletal phenotypes across other organs and tissues

a, Relative expression in osteocytes and 12 non-skeletal tissues (% of maximum expression) of genes with established roles in the osteocyte network **(i)**, and four osteocyte transcriptome signature genes associated with significant skeletal phenotypes in the Origins of Bone and Cartilage Disease Program database **(ii)**. **b,** Normalised expression values of four osteocyte transcriptome signature genes associated with significant skeletal phenotypes in osteocyte-enriched bone and 12 non-skeletal tissues. (n=8 biologically independent mice per samples type). FPKM = Fragments per kilobase per million mapped reads. Data are presented as mean ±SD.

Supplementary Figure 11

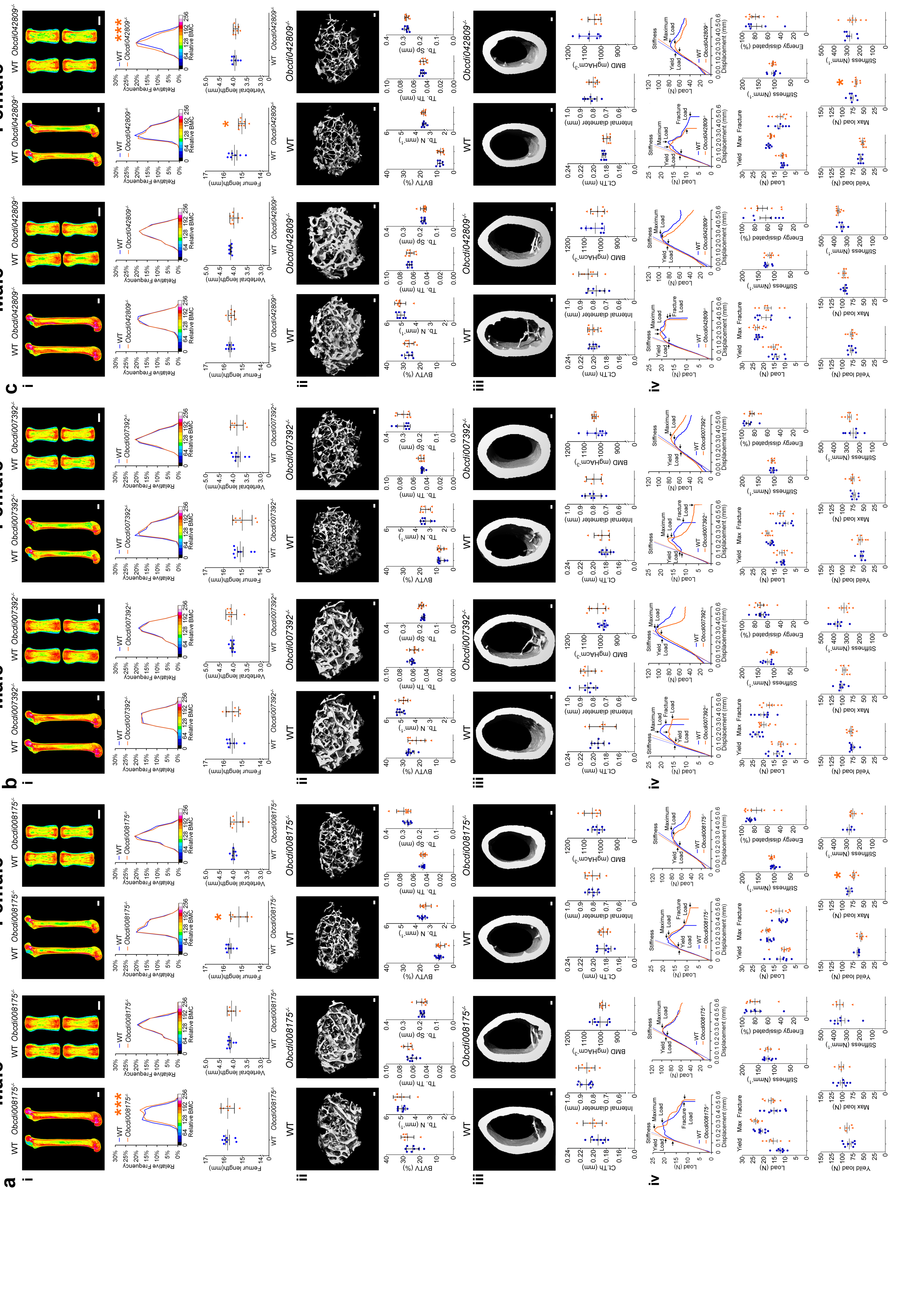

Supplementary Fig. 11: Deletion of novel osteocyte transcriptome signature genes effects skeletal structure and function

Skeletal phenotype of 16-week-old adult mice with deletion of novel genes identified in the osteocyte transcriptome signature. **a,** Mouse line *Obcdi008175*$^{-/-}$ (n=8 WT male, n=6 KO male, n=8 WT female and n=8 KO female). **b,** Mouse line *Obcdi007392*$^{-/-}$ (n=8 WT male, n=8 KO male, n=8 WT female and n=7 KO female) and **c,** Mouse line *Obcdi042809*$^{-/-}$ (n=7 WT male, n=8 KO male, n=7 WT female and n=8 KO female). For each line, data from male mice are shown on the left and data from female mice on the right. Data from wild type animals are shown in blue and data from homozygous knockouts in orange. **(i)** Representative quantitative X-ray microradiographic images from the femurs and vertebrae. Pseudo-coloured images represent grey scale images using a 16-colour interval scheme with low mineral content blue and high mineral content red. Scale bar = 1 mm. Top graphs show relative frequency histograms of femur bone mineral content (BMC) left and vertebra BMC right (***P<0.001 versus WT; Kolmogorov-Smirnov test). Bottom graphs show femur lengths and caudal vertebral heights (mean ± SD), (*P<0.05, versus WT; unpaired Students' *t*-test 2 sided). **(ii)** Representative micro-CT images of distal femur trabecular bone. Scale bar = 100μm. Graphs show bone volume as a proportion of tissue volume (BV/TV), trabecular number (Tb.N), trabecular thickness (Tb.Th), trabecular separation (Tb.Sp) (mean ± SD). **(iii)** Representative micro-CT images of femur cortical bone. Scale bar = 100μm. Graphs show cortical thickness (Ct.Th), internal endosteal diameter and bone mineral density (BMD) (mean ± SD). **(iv)** Representative load displacement curves from 3-point bend testing of the femur (left) and caudal vertebrae compression testing (right). Black arrows indicate yield, maximum and fracture loads and stiffness is indicated by straight blue (WT) or orange (knockout) lines. Top graphs show yield load, maximum and fracture loads, stiffness and energy dissipated prior to fracture (toughness) from femur 3-point bend testing (mean ± SD). Bottom graphs show yield and maximum loads and stiffness from caudal vertebrae compression testing (mean ± SD), (*P<0.05, versus WT; unpaired Students' *t*-test 2 sided).

Supplementary Figure 12

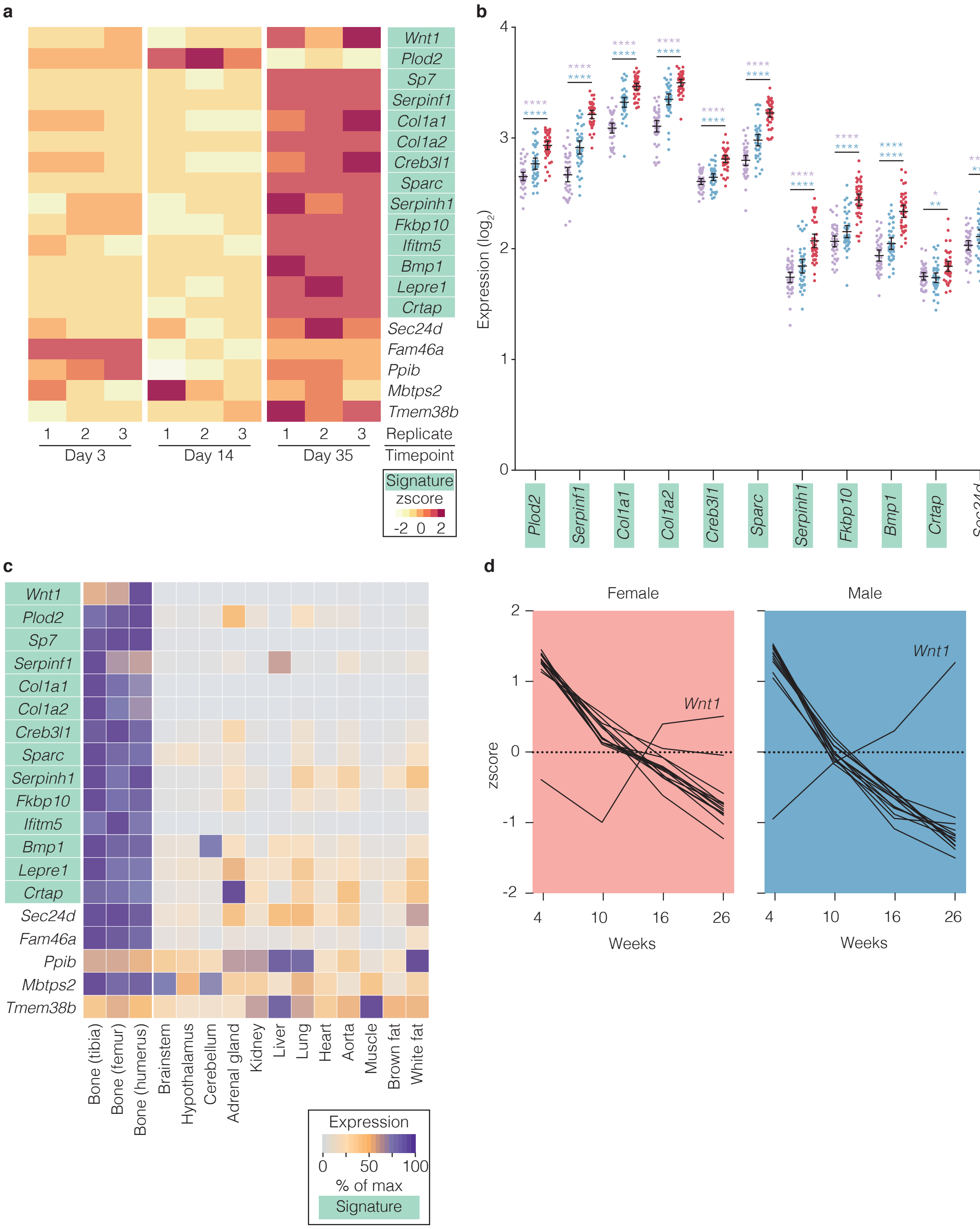

Genes	Female	Male
Wnt1	0.71	0.80
Plod2	-0.18	-0.79
Sp7	-0.74	-0.81
Serpinf1	-0.91	-0.86
Col1a1	-0.87	-0.85
Col1a2	-0.87	-0.86
Creb3l1	-0.80	-0.85
Sparc	-0.91	-0.87
Serpinh1	-0.84	-0.85
Fkbp10	-0.83	-0.86
Ifitm5	-0.60	-0.77
Bmp1	-0.82	-0.89
Lepre1	-0.77	-0.84
Crtap	-0.80	-0.83

Supplementary Fig. 12: Expression of osteogenesis imperfecta genes in the osteocyte transcriptome signature

a, Expression of osteogenesis imperfecta (OI) genes during osteocyte differentiation from osteoblast-like cells (day 3) to early (day 14) and late osteocytes (day 35) (z-score of normalized counts)[50]. Numbers represent replicates. Osteocyte transcriptome signature genes are highlighted in green. **b,** Expression of OI-genes in rat osteocytes (red), osteoblasts (blue) and bone lining cells (purple) isolated by laser capture micro-dissection[16]. 15/19 OI-genes had rat-orthologs on the original microarray and all are shown. Dots represent individual sample expression values (n=40 per cell type). Osteocyte transcriptome signature genes are highlighted in green. Data are presented as mean values +/- 95% confidence interval. P-values were calculated by pairwise t-test (Bonferroni adjusted). Significant differences in expression are indicated by ** ($P<0.01$), *** ($P<0.001$) and **** ($P<0.0001$), with colours denoting comparisons between osteocyte and osteoblasts (blue) and between osteocytes and bone lining cells (purple). **c,** Heatmap showing expression of OI-genes in osteocytes isolated from the tibia, femur and humerus relative to 12 organs and tissues[28] (shown as percentage of maximum mean-FPKM). Osteocyte transcriptome signature genes are highlighted in green. **d,** OI-gene expression in osteocytes with age (4-26 weeks) in female (pink) and male (blue) mice during skeletal maturation. Lines represent individual OI-genes. Pearson correlations between genes and age are tabulated. Genes significantly correlated with age are highlighted (negative = blue, positive = red, $p<0.05$).

Supplementary Table 1 – Short guide RNAs (sgRNAs) to target novel signature genes.

Mouse Line	sgRNAs (PAMs underlined)	Deletion Begin/End	Deletion Size
Obcdi008175$^{-/-}$	AGTACACGCTAATTACTCTC<u>TGG</u><br>CTTGCCTATATGCTGTAAGA<u>AGG</u>	Ch10 107199577<br>Ch10 107205598	6,022 bp
Obcdi007392$^{-/-}$	AGAAATCACCTCACATAGCG<u>AGG</u><br>CCACTTGTAGGCTTAGTGGG<u>CGG</u>	Ch10 56970224<br>Ch10 56998813	28,590 bp
Obcdi042809$^{-/-}$	GTTGGACTTCATCAGAGTTC<u>AGG</u><br>TACTGTAAGAAGGAACGTCA<u>GGG</u>	Ch3 6751276<br>Ch3 6759837	8,562 bp
