# Supplementary material for: Osteocyte transcriptome mapping identifies a molecular landscape controlling skeletal homeostasis and susceptibility to skeletal disease
Source: Nat Commun. 2021 May 5;12:2444. doi: 10.1038/s41467-021-22517-1 (PMC8100170; doi:10.1038/s41467-021-22517-1)
Supplement: Supplementary file 2 — Reporting Summary [file 41467_2021_22517_MOESM2_ESM.pdf]

nature research

Corresponding author(s): Peter I Croucher

Last updated by author(s): Mar 3, 2021

Reporting Summary

Nature Research wishes to improve the reproducibility of the work that we publish. This form provides structure for consistency and transparency in reporting. For further information on Nature Research policies, see our Editorial Policies and the Editorial Policy Checklist.

Statistics

For all statistical analyses, confirm that the following items are present in the figure legend, table legend, main text, or Methods section.

n/a	Confirmed	
☐	☒	The exact sample size (n) for each experimental group/condition, given as a discrete number and unit of measurement
☐	☒	A statement on whether measurements were taken from distinct samples or whether the same sample was measured repeatedly
☐	☒	The statistical test(s) used AND whether they are one- or two-sided<br>*Only common tests should be described solely by name; describe more complex techniques in the Methods section.*
☐	☒	A description of all covariates tested
☐	☒	A description of any assumptions or corrections, such as tests of normality and adjustment for multiple comparisons
☐	☒	A full description of the statistical parameters including central tendency (e.g. means) or other basic estimates (e.g. regression coefficient) AND variation (e.g. standard deviation) or associated estimates of uncertainty (e.g. confidence intervals)
☐	☒	For null hypothesis testing, the test statistic (e.g. F, t, r) with confidence intervals, effect sizes, degrees of freedom and P value noted<br>*Give P values as exact values whenever suitable.*
☒	☐	For Bayesian analysis, information on the choice of priors and Markov chain Monte Carlo settings
☒	☐	For hierarchical and complex designs, identification of the appropriate level for tests and full reporting of outcomes
☐	☒	Estimates of effect sizes (e.g. Cohen's d, Pearson's r), indicating how they were calculated

Our web collection on statistics for biologists contains articles on many of the points above.

Software and code

Policy information about availability of computer code

Data collection	For transcriptome experiments -<br>Bcl2Fastq (v1.8.4)<br>Data from the UK Biobank (https://www.ukbiobank.ac.uk), ArrayExpress (https:// www.ebi.ac.uk/arrayexpress) or Gene Expression Omnibus (GEO, https://www.ncbi.nlm.nih.gov/geo/) were downloaded via their FTP protocols<br><br>For phenotyping experiments -<br>NRecon (Bruker, http://bruker-microct.com/products/downloads.htm)<br>CTAn (Bruker, http://bruker-microct.com/products/downloads.htm)<br>Scanco (v6.4-2)<br>Drishti-2 (v2.6.1, https://github.com/nci/drishti, RRID:SCR_017999)<br>Aperio Imagescope (Leica, https://www.leicabiosystems.com/digital-pathology/manage/aperio-imagescope)<br>ImageJ (https://imagej.net, RRID:SCR_003070)<br>Fiji (http://fiji.sc, RRID:SCR_002285)<br>BoneJ (http://bonej.org, RRID:SCR_018166)<br>Osteomeasure (v3.2.1.8, http://www.osteometrics.com)<br>CTAn, NRecon and Drishti were run using a Windows 7 OS.
Data analysis	Transcriptome analyses using Trinity (v2.0.6), Stringtie (v1.0.4), Cuffcompare (v2.2.1), Trimgalore (v0.3.3), STAR (v2.4.1d) and RSEM (v1.2.21) were performed on a computing cluster running the CentOS 6.8 (Rocks 6.2) Linux operating system.<br><br>GraphPad Prism (v7 and v8, RRID:SCR_002798) was used for statistical analyses and visualization as specific in the methods.<br>Graeber lab online hypergeometric calculator (https://systems.crump.ucla.edu/hypergeometric/index.php), CPAT (v1.2, http://

lilab.research.bcm.edu/cpat) and ReViGo (http://revigo.irb.hr) analyses were run using the web interface.

GWAS analysis was performed using MAGMA (v1.06, https://ctg.cncr.nl/software/magma), LDSC-SEG (v1.1, https://github.com/bulik/ldsc).

R Project for Statistical Computing (>v3.4.0, http://www.r-project.org; RRID:SCR_001905) was used for analyses unless otherwise stated in the methods.
R-packages: biomaRt (v2.34.2), oligo (v1.42.0), affycoretools (v1.50.6), affy (v1.56.0), ggplot2 (v2.2.1), tispec (v0.99.0, https://rdrr.io/github/roonysgalbi/tispec), WGCNA (v1.60), ClusterProfiler (v3.4.4), DOSE (v3.2.0), gplots (v3.0.1), mclust (v5.3), limma (v3.32.7), mixtools (v1.1.0), Pathview (v1.16.5), clues (v0.5.9), Gvis (v1.22.3), ggbio (v1.26.1)

Custom code used to define gene activity, gene expression enrichment in osteocytes and the osteocyte transcriptome signature are available in a public Github repository accessible at https://github.com/scottyoulten/osteocyte_signature

For manuscripts utilizing custom algorithms or software that are central to the research but not yet described in published literature, software must be made available to editors and reviewers. We strongly encourage code deposition in a community repository (e.g. GitHub). See the Nature Research guidelines for submitting code & software for further information.

Data

Policy information about availability of data

All manuscripts must include a data availability statement. This statement should provide the following information, where applicable:

- Accession codes, unique identifiers, or web links for publicly available datasets
- A list of figures that have associated raw data
- A description of any restrictions on data availability

The raw RNA-sequencing data (fastq), read alignment files (BAM) and processed gene expression data files for each cohort (FPKM and counts) are deposited at ArrayExpress (https://www.ebi.ac.uk/arrayexpress) under the following accession numbers: Bone comparison cohort (E-MTAB-5532, https://www.ebi.ac.uk/arrayexpress/experiments/E-MTAB-5532), Skeletal maturation cohort (E-MTAB-7447 , https://www.ebi.ac.uk/arrayexpress/experiments/E-MTAB-7447) and Osteocyte enrichment cohort (E-MTAB-5533, https://www.ebi.ac.uk/arrayexpress/experiments/E-MTAB-5533). There are no restrictions on data availability.

Various publicly available data resources were used in this study: To compare the osteocyte transcriptome to that of other tissues, publicly available sequencing read data was obtained (ArrayExpress accession E-GEOD-54652, https://www.ebi.ac.uk/arrayexpress/experiments/E-GEOD-54652). To determine whether the Magenta Cluster identified genes associated with perilacunar- remodeling, their expression was examined during lactation using a publicly available microarray dataset (ArrayExpress accession E-GEOD-23496, https://www.ebi.ac.uk/arrayexpress/experiments/E-GEOD-23496). To compare expression of osteocyte transcriptome signature genes in osteocyte with other bone-cell types we used a publicly available microarray dataset which profiled gene expression in laser capture micro-dissected osteoblasts, bone-lining cells and osteocytes (GEO accession GSE71306, https://www.ncbi.nlm.nih.gov/geo/query/acc.cgi?acc=GSE71306). To examine the expression of osteocyte transcriptome signature genes during osteocyte differentiation, we analyzed publicly available transcriptome sequencing data from an in vitro model of osteoblast-like cell to osteocyte differentiation (ArrayExpress accession E-GEOD-54783, https://www.ebi.ac.uk/arrayexpress/experiments/E-GEOD-54783). Human genotype and phenotype data on which the results of this study were based were accessed from the UK Biobank (http://www.ukbiobank.ac.uk/) with accession ID 53641.

Field-specific reporting

Please select the one below that is the best fit for your research. If you are not sure, read the appropriate sections before making your selection.

[x] Life sciences [ ] Behavioural & social sciences [ ] Ecological, evolutionary & environmental sciences

For a reference copy of the document with all sections, see nature.com/documents/nr-reporting-summary-flat.pdf

Life sciences study design

All studies must disclose on these points even when the disclosure is negative.

Sample size

For transcriptome experiments, power analysis was conducted using the Scotty webtool (http://scotty.genetics.utah.edu/). Pilot RNA sequencing data was used to assess the amount of biological variance. Analysis indicated 5 biological replicates sequenced to a depth of 20 million reads was sufficient to detect ~80% of genes with 2-fold change in expression at p<0.05. All experiments match or exceed these specification, either in replicate number, sequencing depth, or both.

For phenotype data associated with transcriptome sequencing studies, sample size was limited to concordant samples taken from contralateral limbs of those used for RNAseq. For the bone comparison study n=8, for skeletal maturation study n=5. For TRAP staining in osteocyte enriched or marrow containing bone samples an n=4 was chosen as pilot studies had shown an absence of cells lining bone in osteocyte enriched samples.

For skeletal phenotyping of knockout mice in the OBCD pipeline the reference ranges for each skeletal parameter were derived from 320 female 16 week old C57BL/6NTac wild-type mice. Using these data together with coefficients of variation for each test, power calculations indicate an 80% power to detect outlier phenotype of greater or equal to 2SD with a sample size of n=2. The exact number of biological replicates examined for each knockout mice line is listed in Supplementary Data 8.

GWAS data from the UK biobank were selected based on stringent quality control criteria to select 362,924 participants. Participants were selected if they had high-quality quantitative heel ultrasound data and if they were of a White British genetic ethnicity. These sample sizes represent the largest sample size to-date for any musculoskeletal trait.

Data exclusions	No data was excluded from transcriptome sequencing studies. Similarly in mouse knockout studies, data for all mice were included, including those with and without skeletal phenotypes.
Replication	Experiments were conducted using multiple independent biological replicates as outlines in the 'Sample size' section. Individual experiments were not replicated directly. Where available, orthogonal, independently produced datasets were used to validate experimental findings.
Randomization	Samples used in transcriptome sequencing were specifically bred for the purposes of this study. Samples collected for transcriptome sequencing in each of the Bone Comparison or Osteocyte Enrichment mouse cohorts were all collected in a single batch. To generate samples for the Skeletal Maturation cohort, breeding was stratified so all samples (representing multiple ages) could be collected within a single 36-hour time period. Samples were collected in groups of 8 mice (one from each time point, in each sex) to avoid confounding batch effects. For rapid throughput phenotyping of knockout mouse lines, samples were anonymized and randomly assigned to batches for analysis.
Blinding	For transcriptome experiments data collection was stratified by sample type to avoid batch effects and thus blinding was not possible. For transcriptome analysis sample type was used as a variable of interest so blinding was not possible. Morphological analysis of samples collected from the contralateral limb of those taken for transcriptome analysis was performed in a blinded manner.<br>For phenotypic analysis, all data collection and analysis was performed in a blinded manner.

Reporting for specific materials, systems and methods

We require information from authors about some types of materials, experimental systems and methods used in many studies. Here, indicate whether each material, system or method listed is relevant to your study. If you are not sure if a list item applies to your research, read the appropriate section before selecting a response.

Materials & experimental systems

n/a	Involved in the study
☒	☐ Antibodies
☒	☐ Eukaryotic cell lines
☒	☐ Palaeontology and archaeology
☐	☒ Animals and other organisms
☐	☒ Human research participants
☒	☐ Clinical data
☒	☐ Dual use research of concern

Methods

n/a	Involved in the study
☒	☐ ChIP-seq
☒	☐ Flow cytometry
☒	☐ MRI-based neuroimaging

Animals and other organisms

Policy information about studies involving animals; ARRIVE guidelines recommended for reporting animal research

Laboratory animals	Bone Comparison cohort: 16-week-old male C57BL6/NTac mice; Skeletal maturation cohort: 4, 10, 16 and 26-week-old female and male C57BL6/NTac mice; Osteocyte Enrichment cohort: 10-week-old male C57BL6/NTac mice.<br>Novel-gene knockout mice lines were produced by CRISPR/Cas9 gene targeting in C57BL/6J mouse embryos. Phenotyping was performed in 16-week-old male and female mice.<br>Animal holding areas were maintained within a constant temperature range of 21-22 degrees Celsius and 60-65% humidity to avoid animal stress and to minimise experiment variability. Lighting in animal rooms ensured 12 hours' light and 12 hours' darkness with a dawn / dusk simulation.<br>Knockout mouse lines screened in the OBCD phenotyping pipeline were produced on a background of C57BL6/NTac mice, with phenotyping experiments conducted on 16-week-old female mice.
Wild animals	The study did not involve wild animals.
Field-collected samples	The study did not involve animals from the field.
Ethics oversight	Animal experiments associated with the Bone Comparison, Skeletal Maturation and Osteocyte Enrichment cohorts were approved by the The Garvan/St Vincent's Animal Ethics Committee (Protocol ID 16/01 and 12/44).<br>Generation of novel-gene knockout mice was approved by the Garvan/St Vincent's Animal Ethics Committee (Protocol ID 18/36).<br>The OBCD mouse studies were undertaken by Wellcome Trust Sanger Institute Mouse Genetics Project (MGP) as part of the IMPC. This was licensed by the UK Home Office (PPLs 80/2485 and P77453634) in accordance with the 1986 Animals (Scientific Procedures) Act and approved by the Wellcome Sanger Institute's Animal Welfare and Ethical Review Body.

Note that full information on the approval of the study protocol must also be provided in the manuscript.

Human research participants

Policy information about studies involving human research participants

Population characteristics	Genetic associations with bone mineral density (eBMD) in the human population were analysed using the UK Biobank Resource (accession ID: 53641). Analyses of eBMD were performed on a sample of 362,924 unrelated white British subjects (54% female) from the UK Biobank Study (UKBB) that had valid quantitative eBMD and high-quality genome-wide HRC and 1000G/UK10K imputed data from the January 2018 release. Analyses of individuals with OA were performed on published,

	precomputed summary statistics from a recent UKBB and arcOGEN GWAS meta-analysis (77,052 cases and 378,169 controls). arcOGEN is a collection of unrelated, UK-based individuals of European ancestry with knee and/or hip osteoarthritis from the arcOGEN Consortium.
Recruitment	No research participants were directly recruited for this work. Access to the anonymised, deidentified data through the UKBB was granted with accession ID: 53641. The UKBB is a cohort of 500,000 participants 40–69 years of age recruited between 2006 and 2010 in 22 assessment centers throughout the UK. Participants completed electronic signed consent, a self-completed touch-screen questionnaire, a brief computer-assisted interview, physical and functional measures, and collection of biological samples and genetic data.
Ethics oversight	Ethical approval for the collection of data for the UKBB data was obtained from the Northwest Multi-Centre Research Ethics Committee and informed consent was obtained from all participants prior to participation. The participants have agreed to share their deidentified data for research purposes. Details of the ethics and governance framework, for the UK Biobank resource can be found at https://www.ukbiobank.ac.uk/media/0xsbmfmw/egf.pdf. UKBB approved this project through accession ID: 53641.<br><br>The arcOGEN study was ethically approved by appropriate review committees, and the prospective collections were approved by the National Research Ethics Service in the United Kingdom. All subjects in arcOGEN provided written, informed consent.

Note that full information on the approval of the study protocol must also be provided in the manuscript.
