# Supplementary material for: Osteocyte transcriptome mapping identifies a molecular landscape controlling skeletal homeostasis and susceptibility to skeletal disease
Source: Nat Commun. 2021 May 5;12:2444. doi: 10.1038/s41467-021-22517-1 (PMC8100170; doi:10.1038/s41467-021-22517-1)
Supplement: Supplementary file 3 — Description of Additional Supplementary Files [file 41467_2021_22517_MOESM3_ESM.docx]

**Supplementary Data Descriptions and Columns**

**Supplementary Data 1.** Sample specific gene activity thresholds

**Description:** The sample specific thresholds of gene activity calculated based on the distribution of normalized gene expression in each sample. Genes that were expressed above the sample specific threshold in all replicates of a given sample type were considered active in that sample type.

**Columns:**

Cohort: Mouse transcriptome sequencing cohort within which the sample was collected.

Sample_ID: Unique sample identifier, corresponds with read count/FPKM data. Labels follow a Type_replicate convention.

Sample_details: Details on sample bone type, processing procedure, sex and age.

Activity_threshold_FPKM: Sample specific gene expression threshold expressed in fragments per kilobase per million mapped reads (FPKM).

**Supplementary Data 2.** The active osteocyte transcriptome

**Description:** Master table summarizing and annotating transcriptome data collected from the *Bone Comparison*, *Skeletal Maturation* and *Osteocyte Enrichment* cohorts

**Columns:**

Ensembl_ID: Mouse Ensembl ID

GeneSymbol: Mouse gene symbol

Description: MGI gene description

EntrezID: Mouse Entrez ID

Biotype: Gene biotype

Human_Ensembl_ID: Orthologous human Ensembl ID

Human_GeneSymbol: Orthologous human gene symbol

Human_Entrez_ID: Orthologous human Entrez ID

Tibia_activity: Number of replicates expressed above sample specific activity threshold (max 8) in tibia samples

Tibia_mean_FPKM: Mean normalised expression (FPKM) of gene across replicates of osteocyte-isolated samples from the bone comparison cohort

Femur_activity: Number of replicates expressed above sample specific activity threshold (max 8) in femur samples

Femur_mean_FPKM: Mean normalised expression (FPKM) of gene across replicates of osteocyte-isolated samples from the bone comparison cohort

Humerus_activity: Number of replicates expressed above sample specific activity threshold (max 8) in humerus samples

Humerus_mean_FPKM: Mean normalised expression (FPKM) of gene across replicates of osteocyte-isolated samples from the bone comparison cohort

Active_tissue_number: Number of non-skeletal organs and tissues in which gene is expressed (max 12). Publicly available data for this was published by PMID25349387

InVitro_active_osteocyte: Genes expressed in either early or late osteocytic IDGSW3 cells during differentiation. Publicly available data used was published by PMID24877565

LaserCaptured_active_osteocyte: Genes expressed in laser capture micro-dissected osteocytes. Publicly available data used was published by PMID26721737

CollagenaseDigested_active_osteocyte: Genes expressed in osteocyte samples that have marrow removed by flushing and centrifugation and then collagenase digestion. Publicly available data used was published by PMID22308018

Female4_activity: Number of replicates expressed above sample specific activity threshold (max 5) in the humeri of 4 week-old female samples

Female4_mean_FPKM: Mean normalised expression (FPKM) of gene across replicates of osteocyte-isolated samples from the skeletal maturation cohort

Female10_activity: Number of replicates expressed above sample specific activity threshold (max 5) in the humeri of 10 week-old female samples

Female10_mean_FPKM: Mean normalised expression (FPKM) of gene across replicates of osteocyte-isolated samples from the skeletal maturation cohort

Female16_activity: Number of replicates expressed above sample specific activity threshold (max 5) in the humeri of 16 week-old female samples

Female16_mean_FPKM: Mean normalised expression (FPKM) of gene across replicates of osteocyte-isolated samples from the skeletal maturation cohort

Female26_activity: Number of replicates expressed above sample specific activity threshold (max 5) in the humeri of 26 week-old female samples

Female26_mean_FPKM: Mean normalised expression (FPKM) of gene across replicates of osteocyte-isolated samples from the skeletal maturation cohort

Male4_activity: Number of replicates expressed above sample specific activity threshold (max 5) in the humeri of 4 week-old male samples

Male4_mean_FPKM: Mean normalised expression (FPKM) of gene across replicates of osteocyte-isolated samples from the skeletal maturation cohort

Male10_activity: Number of replicates expressed above sample specific activity threshold (max 5) in the humeri of 10 week-old male samples

Male10_mean_FPKM: Mean normalised expression (FPKM) of gene across replicates of osteocyte-isolated samples from the skeletal maturation cohort

Male16_activity: Number of replicates expressed above sample specific activity threshold (max 5) in the humeri of 16 week-old male samples

Male16_mean_FPKM: Mean normalised expression (FPKM) of gene across replicates of osteocyte-isolated samples from the skeletal maturation cohort

Male26_activity: Number of replicates expressed above sample specific activity threshold (max 5) in the humeri of 26 week-old male samples

Male26_mean_FPKM: Mean normalised expression (FPKM) of gene across replicates of osteocyte-isolated samples from the skeletal maturation cohort

Skeletal_maturation_cluster: WGCNA cluster to which gene was assigned based on expression in the skeletal maturation cohort (denoted by colors)

Skeletal_maturation_cluster_corr: Correlation with assigned cluster eigengene

Skeletal_maturation_cluster_corr_pval: P-value of correlation with assigned cluster eigengene

Osteocyte_activity: Number of replicates expressed above sample specific activity threshold (max 5)

Osteocyte_mean_FPKM: Mean normalised expression (FPKM) of gene across replicates of osteocyte-isolated samples from the Osteocyte Enrichment cohort

Osteocyte_marrow_activity: Number of replicates expressed above sample specific activity threshold (max 5)

Osteocyte_marrow_mean_FPKM: Mean normalised expression (FPKM) of gene across replicates of whole-bone samples from the Osteocyte Enrichment cohort

LFC: Log2 fold-change in expression data between osteocyte-isolated and whole-bone samples from the Osteocyte Enrichment cohort

LFC_CI_Upper: Upper 95% confidence interval of LFC

LFC_CI_Lower: Lower 95% confidence interval of LFC

Above_threshold: Gene LFC_CI_Lower above osteocyte enrichment threshold (TRUE/FALSE)

Ayturk_muscle: Genes significantly enriched in skeletal muscle relative to osteocyte-enriched bone samples. Publicly available data used was published by PMID23553928

Ayturk_marrow: Genes significantly enriched in bone marrow relative to osteocyte-enriched bone samples. Publicly available data used was published by PMID23553928

Ayturk_blood: Genes significantly enriched in blood relative to osteocyte-enriched bone samples. Publicly available data used was published by PMID23553928

Ayturk_contaminant: Gene enriched in either Ayturk_muscle, Ayturk_marrow, Ayturk_blood.

Osteocyte_transcriptome: Genes active osteocytes from any sample type in either the bone comparison cohort, the skeletal maturation cohort or osteocyte enrichment cohort

Osteocyte_transcriptome_signature: Genes identified in the osteocyte transcriptome signature (Osteocyte_transcriptome = TRUE & Above_threshold = TRUE & Ayturk_contaminant = FALSE)

Total_gene_universe: Genes active in the Osteocyte_transcriptome or any of the 12 non-skeletal tissues assayed in PMID25349387

Osteocyte_Tau: Tissue specificity index calculated relative to 12 non-skeletal tissues assayed in PMID25349387

Skeletal_GO_Term: Gene ontology term IDs associated with gene that are related to skeletal biological processes

Skeletal_MP_Term: Genes associated with skeletal mammalian phenotype terms in the MGI database

Skeletal_annotation: Genes annotated with either Skeletal_GO_Term or Skeletal_MP_Term

IDGSW3_Day3_mean_CPM: Mean normalised expression (CPM) of gene across replicates of IDGSW3 cells at day 3 of differentiation. Publicly available data used was published by PMID24877565

IDGSW3_Day14_mean_CPM: Mean normalised expression (CPM) of gene across replicates of IDGSW3 cells at day 14 of differentiation. Publicly available data used was published by PMID24877565

IDGSW3_Day35_mean_CPM: Mean normalised expression (CPM) of gene across replicates of IDGSW3 cells at day 35 of differentiation. Publicly available data used was published by PMID24877565

IDGSW3_cluster: Cluster to which osteocyte transcriptome signature gene is assigned based on expression in IDGSW3 cells during osteocytic differentiation

OBCD_phenotype: Osteocyte transcriptome signature genes associated with significant skeletal phenotypes in the OBCD pipeline

**Supplementary Data 3.** Osteocyte transcriptome genes differentially expressed between bones

**Description:** Genes differentially expressed in osteocytes between skeletal sites in the *Bone Comparison* Cohort.

**Columns:**

Ensembl_ID: Mouse Ensembl ID

GeneSymbol: Mouse gene symbol

Description: MGI gene description

EntrezID: Mouse Entrez ID

Comparison: Sample types used in differential expression test between Tibia (Tib), Femur (Fem) or Humerus (Hum). (Sample1-Sample2)

logFC: Log2 fold-change of expression in Sample1 relative to Sample2.

t: T-statistic associated with logFC of Comparison

adj.P.Val: BH-adjuested p-value associated with logFC of Comparison

Tibia_activity: Number of replicates expressed above sample specific activity threshold (max 8) in tibia samples

Tibia_mean_FPKM: Mean normalised expression (FPKM) of gene across replicates of osteocyte-isolated samples from the bone comparison cohort

Femur_activity: Number of replicates expressed above sample specific activity threshold (max 8) in femur samples

Femur_mean_FPKM: Mean normalised expression (FPKM) of gene across replicates of osteocyte-isolated samples from the bone comparison cohort

Humerus_activity: Number of replicates expressed above sample specific activity threshold (max 8) in humerus samples

Humerus_mean_FPKM: Mean normalised expression (FPKM) of gene across replicates of osteocyte-isolated samples from the bone comparison cohort

**Supplementary Data 4.** Skeletal maturation clusters

**Description:** Clusters of genes with correlated patterns of expression in the *Skeletal Maturation* cohort (sheets are named by cluster color).

**Columns:**

Ensembl_ID: Mouse Ensembl ID

GeneSymbol: Mouse gene symbol

Description: MGI gene description

EntrezID: Mouse Entrez ID

Biotype: Gene biotype

Female4_activity: Number of replicates expressed above sample specific activity threshold (max 5) the humeri of 4 week-old female samples

Female4_mean_FPKM: Mean normalised expression (FPKM) of gene across replicates of osteocyte-isolated samples from the skeletal maturation cohort

Female10_activity: Number of replicates expressed above sample specific activity threshold (max 5) the humeri of 10 week-old female samples

Female10_mean_FPKM: Mean normalised expression (FPKM) of gene across replicates of osteocyte-isolated samples from the skeletal maturation cohort

Female16_activity: Number of replicates expressed above sample specific activity threshold (max 5) the humeri of 16 week-old female samples

Female16_mean_FPKM: Mean normalised expression (FPKM) of gene across replicates of osteocyte-isolated samples from the skeletal maturation cohort

Female26_activity: Number of replicates expressed above sample specific activity threshold (max 5) the humeri of 26 week-old female samples

Female26_mean_FPKM: Mean normalised expression (FPKM) of gene across replicates of osteocyte-isolated samples from the skeletal maturation cohort

Male4_activity: Number of replicates expressed above sample specific activity threshold (max 5) the humeri of 4 week-old male samples

Male4_mean_FPKM: Mean normalised expression (FPKM) of gene across replicates of osteocyte-isolated samples from the skeletal maturation cohort

Male10_activity: Number of replicates expressed above sample specific activity threshold (max 5) the humeri of 10 week-old male samples

Male10_mean_FPKM: Mean normalised expression (FPKM) of gene across replicates of osteocyte-isolated samples from the skeletal maturation cohort

Male16_activity: Number of replicates expressed above sample specific activity threshold (max 5) the humeri of 16 week-old male samples

Male16_mean_FPKM: Mean normalised expression (FPKM) of gene across replicates of osteocyte-isolated samples from the skeletal maturation cohort

Male26_activity: Number of replicates expressed above sample specific activity threshold (max 5) the humeri of 26 week-old male samples

Male26_mean_FPKM: Mean normalised expression (FPKM) of gene across replicates of osteocyte-isolated samples from the skeletal maturation cohort

Skeletal_maturation_cluster: WGCNA cluster to which gene was assigned based on expression in the skeletal maturation cohort (denoted by colors)

Skeletal_maturation_cluster_corr: Correlation with assigned cluster eigengene

Skeletal_maturation_cluster_corr_pval: P-value of correlation with assigned cluster eigengene

**Supplementary Data 5.** The *osteocyte transcriptome signature*

**Description:** Annotated list of genes in the *osteocyte transcriptome signature* with summary expression information.

**Columns:**

Column descriptions are the same as **Supplementary Data 1**

**Supplementary Data 6.** Novel genes expressed in osteocytes

**Description:** Description and sequence of novel genes revealed by *de novo* transcriptome assembly of osteocyte transcriptome data.

**Columns:**

Gene_ID: Gene ID assigned to assembled loci. Obcda idenitify loci located the antisense strand to known genes. Obcdi identify loci located in intergenic regions

Transcript_ID: Transcript ID assigned to unique transcripts assembled at each loci

Chr: Chromosome location of assembled loci

Start_bp: Base-pair start point of transcript

End_bp: Base-pair end point of transcript

Exon_count: Number of exons in assembled transcript

Coding_potential: Coding potential of assembled transcript

Antisense_gene_symbol: Gene symbol of gene located on antisense strand of novel loci (for Obcda genes)

Tibia_activity: Number of replicates expressed above sample specific activity threshold (max 8) in tibia samples (gene level)

Tibia_mean_FPKM: Mean normalised expression (FPKM) of gene across replicates of osteocyte-isolated samples from the bone comparison cohort

Femur_activity: Number of replicates expressed above sample specific activity threshold (max 8) in femur samples (gene level)

Femur_mean_FPKM: Mean normalised expression (FPKM) of gene across replicates of osteocyte-isolated samples from the bone comparison cohort

Humerus_activity: Number of replicates expressed above sample specific activity threshold (max 8) in humerus samples (gene level)

Humerus_mean_FPKM: Mean normalised expression (FPKM) of gene across replicates of osteocyte-isolated samples from the bone comparison cohort

Osteocyte_transcriptome: Genes active osteocytes from any sample type in either the bone comparison cohort, the skeletal maturation cohort or osteocyte enrichment cohort

Osteocyte_transcriptome_signature: Genes identified in the osteocyte transcriptome signature

Transcript_sequence: Base-pair sequence of assembled transcript

**Supplementary Data 7.** Gene ontology terms enriched in the *osteocyte transcriptome signature*

**Description:** Genes and enrichment statistics associated with GO biological processes significantly enriched among *osteocyte transcriptome signature* genes.

**Columns:**

GO_Term_ID: GO term IDs

GO_Description: GO term descriptions

Semantic_cluster_classification: Semantically related cluster to which GO_term was assigned

log10.p.value: Bonferroni corrected p-value of GO-term enrichment among osteocyte transcriptome signature genes

Genes: Osteocyte transcriptome signature genes associated with GO-term

**Supplementary Data 8.** *Osteocyte transcriptome signature* genes deleted in mice and phenotyped by the origins of bone and cartilage disease program

**Description**: Phenotyping data for the 64 *osteocyte transcriptome signature* genes screened by the OBCD functional skeletal phenotyping pipeline (sheet 1). Annotation of 26 *osteocyte transcriptome signature* genes associated with significant skeletal phenotypes in OBCD pipeline. Genes represent in **Fig. 5b-d** are colored concordantly (sheet 2).

**Columns:**

Ensembl ID: Mouse Ensembl ID

Mouse Gene: Mouse gene symbol target of knockout allele

Human Gene: Human ortholog symbol of Mouse Gene

Gene Name: Mouse Gene name

GO: Biological processes and molecular function listed in the GO database to which the mouse gene is annotated

Genotype: Genotype at knockout allele

Allele type: Mouse knockout allele

n: Number of mice with knockout allele screened through pipeline

Femur analysis: Morphological and functional phenotyping parameters derived from measurements of the femur. Values represent standard deviations away from mean of wild-type mouse reference range

Vertebral analysis: Morphological and functional phenotyping parameters derived from measurements of the vertebrae. Values represent standard deviations away from mean of wild-type mouse reference range

BMC: Bone mineral content

Length: Bone length

BV/TV: Bone tissue volume as a fraction of total tissue volume

Tb.N: Trabecular number

Tb.Th: Trabecular thickness

Tb.Sp: Trabecular spacing

Cort.Th: Cortical thickness

Internal Diameter: Internal cortical diameter

Bone Density: Bone mineral density

Yield Load: Bone yield load prior to fracture

Max Load: Bone maximum load prior to fracture

Fracture Load: Bone fracture load

Stiffness: Bone stiffness

DE Fracture: Dissipated energy prior to fracture

**Supplementary Data 9.** Expression of genetic skeletal disorder genes in the osteocyte network

**Description:** Annotation of osteocyte expression and enrichment for genes known to cause human skeletal dysplasia.

**Columns:**

Human_Ensembl_ID: Orthologous human Ensembl ID

Human_GeneSymbol: Orthologous human gene symbol

Human_Entrez_ID: Orthologous human Entrez ID

Nosology_ID: Unique idenitifier assigned to nosology disease group (defined in PMID:31633310)

Group: Name of nosology defined disease group

Disease: Name of disease associted with gene

Mouse_Ensembl_ID: Mouse Ensembl ID

Mouse_GeneSymbol: Mouse gene symbol

Mouse_EntrezID: Mouse Entrez ID

Description: MGI gene description

Skeletal_GO_Term: Gene ontology term IDs associated with gene that are related to skeletal biological processes

Skeletal_MP_Term: Genes associated with skeletal mammalian phenotype terms in the MGI database

Skeletal_annotation: Genes annotated with either Skeletal_GO_Term or Skeletal_MP_Term

Osteocyte_transcriptome: Genes active osteocytes from any sample type in either the bone comparison cohort, the skeletal maturation cohort or osteocyte enrichment cohort

Osteocyte_transcriptome_signature: Genes identified in the osteocyte transcriptome signature

Female4_activity: Number of replicates expressed above sample specific activity threshold (max 5) the humeri of 4 week-old female samples

Female4_mean_FPKM: Mean normalised expression (FPKM) of gene across replicates of osteocyte-isolated samples from the skeletal maturation cohort

Female10_activity: Number of replicates expressed above sample specific activity threshold (max 5) the humeri of 10 week-old female samples

Female10_mean_FPKM: Mean normalised expression (FPKM) of gene across replicates of osteocyte-isolated samples from the skeletal maturation cohort

Female16_activity: Number of replicates expressed above sample specific activity threshold (max 5) the humeri of 16 week-old female samples

Female16_mean_FPKM: Mean normalised expression (FPKM) of gene across replicates of osteocyte-isolated samples from the skeletal maturation cohort

Female26_activity: Number of replicates expressed above sample specific activity threshold (max 5) the humeri of 26 week-old female samples

Female26_mean_FPKM: Mean normalised expression (FPKM) of gene across replicates of osteocyte-isolated samples from the skeletal maturation cohort

Male4_activity: Number of replicates expressed above sample specific activity threshold (max 5) the humeri of 4 week-old male samples

Male4_mean_FPKM: Mean normalised expression (FPKM) of gene across replicates of osteocyte-isolated samples from the skeletal maturation cohort

Male10_activity: Number of replicates expressed above sample specific activity threshold (max 5) the humeri of 10 week-old male samples

Male10_mean_FPKM: Mean normalised expression (FPKM) of gene across replicates of osteocyte-isolated samples from the skeletal maturation cohort

Male16_activity: Number of replicates expressed above sample specific activity threshold (max 5) the humeri of 16 week-old male samples

Male16_mean_FPKM: Mean normalised expression (FPKM) of gene across replicates of osteocyte-isolated samples from the skeletal maturation cohort

Male26_activity: Number of replicates expressed above sample specific activity threshold (max 5) the humeri of 26 week-old male samples

Male26_mean_FPKM: Mean normalised expression (FPKM) of gene across replicates of osteocyte-isolated samples from the skeletal maturation cohort

Skeletal_maturation_cluster: WGCNA cluster to which gene was assigned based on expression in the skeletal maturation cohort (denoted by colors)

Skeletal_maturation_cluster_corr: Correlation with assigned cluster eigengene

Skeletal_maturation_cluster_corr_pval: P-value of correlation with assigned cluster eigengene

Tibia_activity: Number of replicates expressed above sample specific activity threshold (max 8) in tibia samples

Tibia_mean_FPKM: Mean normalised expression (FPKM) of gene across replicates of osteocyte-isolated samples from the bone comparison cohort

Femur_activity: Number of replicates expressed above sample specific activity threshold (max 8) in femur samples

Femur_mean_FPKM: Mean normalised expression (FPKM) of gene across replicates of osteocyte-isolated samples from the bone comparison cohort

Humerus_activity: Number of replicates expressed above sample specific activity threshold (max 8) in humerus samples

Humerus_mean_FPKM: Mean normalised expression (FPKM) of gene across replicates of osteocyte-isolated samples from the bone comparison cohort

Osteocyte_activity: Number of replicates expressed above sample specific activity threshold (max 5)

Osteocyte_mean_FPKM: Mean normalised expression (FPKM) of gene across replicates of osteocyte-isolated samples from the Osteocyte Enrichment cohort

Osteocyte_marrow_activity: Number of replicates expressed above sample specific activity threshold (max 5)

Osteocyte_marrow_mean_FPKM: Mean normalised expression (FPKM) of gene across replicates of whole-bone samples from the Osteocyte Enrichment cohort

LFC: Log2 fold-change in expression data between osteocyte-isolated and whole-bone samples from the Osteocyte Enrichment cohort

LFC_CI_Upper: Upper 95% confidence interval of LFC

LFC_CI_Lower: Lower 95% confidence interval of LFC

Above_threshold: Gene LFC_CI_Lower above osteocyte enrichment threshold (TRUE/FALSE)

**Supplementary Data 10.** *Osteocyte transcriptome signature* genes associated with eBMD in the UK Biobank cohort

**Description:** *Osteocyte transcriptome signature* genes significantly associated with eBMD based on gene-wise tests of enrichment (sheet 1). Genes from sheet 1 that are known to cause skeletal dysplasia when mutated in humans (sheet 2). The nearest genes to conditionally independent GWAS variants significantly associated with eBMD and their identification in the osteocyte signature (sheet 3).

**Columns (sheets 1 and 2):**

Mouse_Ensembl_ID: Mouse Ensembl ID

Human_Ensembl_ID: Orthologous human Ensembl ID

Human_GeneSymbol: Orthologous human gene symbol

Human_Entrez_ID: Orthologous human Entrez ID

CHR: Chromosomal location of human ortholog (hg19)

START: Gene start base-pair

STOP: Gene end base-pair

NPARAM: the number of relevant parameters used in the model. For the SNP-wise models this is an approximate value when using the ‘sum’ model (--pval default) or simply the number of SNPs otherwise; for the principal components regression this is set to the number of principal components retained after pruning

N: the sample size used when analysing that gene; can differ for allosomal chromosomes or when analysing SNP p-value input with variable sample size by SNP (due to missingness or differences in coverage in meta-analysis)

ZSTAT: the Z-value for the gene, based on its (permutation) p-value; this is what is used as the measure of gene association in the gene-level analyses

P_SNPWISE_MEAN: Mean p-value for SNPs in gene

P_SNPWISE_TOP1: Highest p-value of a SNP in gene

P_PCREG: Principal component corrected p-value

P_JOINT: Joint p-value of gene-eBMD association (used to identify significantly associated genes)

Skeletal_GO_Term: Gene ontology term IDs associated with gene that are related to skeletal biological processes

Skeletal_MP_Term: Genes associated with skeletal mammalian phenotype terms in the MGI database

OBCD_phenotype: Osteocyte transcriptome signature genes associated with significant skeletal phenotypes in the OBCD pipeline

LFC: Log2 fold-change in expression data between osteocyte-isolated and whole-bone samples from the Osteocyte Enrichment cohort

Skeletal_dysplasia: Genes known to cause skeletal dysplasia when mutated in humans

Nearest_gene: Nearest gene to conditionally independent GWAS variants significantly associated with eBMD

**Columns (sheet 3):**

SNPID: ID of lead conditionally independent GWAS variants significantly associated with eBMD

RSID: RSID of conditionally independent GWAS variants significantly associated with eBMD

CHR: Chromosome location of conditionally independent GWAS variants significantly associated with eBMD

BP: base-pair location of conditionally independent GWAS variants significantly associated with eBMD

C.GENE: Human gene symbol of gene nearest to lead conditionally independent GWAS variants significantly associated with eBMD

C.DIST: Distance of gene nearest to the lead conditionally independent GWAS variants significantly associated with eBMD

Human_ensembl_ID: Human Ensembl ID of C.GENE

Mouse_Ensembl_ID: Orthologous mouse Ensembl ID to Human_ensembl_ID

Universe: Gene identified in gene universe as defined in methods section ‘*Enrichment of osteocyte transcriptome signature for orthologs associated with Osteoporosis and OA’*

Osteocyte_signature: Mouse ortholog identified in the *osteocyte transcriptome signature*

LFC: Log2 fold-change in expression data between osteocyte-isolated and whole-bone samples from the *Osteocyte Enrichment* cohort

Gene-level_significant: Genes significantly associated with eBMD based on gene-wise tests of enrichment (sheet 1)

**Supplementary Data 11.** *Osteocyte transcriptome signature* genes associated with OA in the UK Biobank cohort

**Description:** Osteocyte transcriptome signature genes significantly associated with OA based on gene-wise tests of enrichment (sheet 1). Genes from sheet 1 that are known to cause skeletal dysplasia when mutated in humans (sheet 2). The nearest genes to conditionally independent GWAS variants significantly associated with OA and their identification in the osteocyte signature (sheet 3).

**Columns (sheets 1 and 2):**

Mouse_Ensembl_ID: Mouse Ensembl ID

Human_Ensembl_ID: Orthologous human Ensembl ID

Human_GeneSymbol: Orthologous human gene symbol

Human_Entrez_ID: Orthologous human Entrez ID

CHR: Chromosomal location of human ortholog (hg19)

START: Gene start base-pair

STOP: Gene end base-pair

NPARAM: the number of relevant parameters used in the model. For the SNP-wise models this is an approximate value when using the ‘sum’ model (--pval default) or simply the number of SNPs otherwise; for the principal components regression this is set to the number of principal components retained after pruning

N: the sample size used when analysing that gene; can differ for allosomal chromosomes or when analysing SNP p-value input with variable sample size by SNP (due to missingness or differences in coverage in meta-analysis)

ZSTAT: the Z-value for the gene, based on its (permutation) p-value; this is what is used as the measure of gene association in the gene-level analyses

P_SNPWISE_MEAN: Mean p-value for SNPs in gene

P_SNPWISE_TOP1: Highest p-value of a SNP in gene

P_PCREG: Principal component corrected p-value

P_JOINT: Joint p-value of gene-OA association (used to identify significantly associated genes)

Dataset: OA-subgroup from which statistics were calculated (defined in PMID:30664745)

Skeletal_GO_Term: Gene ontology term IDs associated with gene that are related to skeletal biological processes

Skeletal_MP_Term: Genes associated with skeletal mammalian phenotype terms in the MGI database

OBCD_phenotype: *Osteocyte transcriptome signature* genes associated with significant skeletal phenotypes in the OBCD pipeline

LFC: Log2 fold-change in expression data between osteocyte-isolated and whole-bone samples from the Osteocyte Enrichment cohort

Skeletal_dysplasia: Genes known to cause skeletal dysplasia when mutated in humans

Nearest_gene: Nearest gene to conditionally independent GWAS variants significantly associated with OA

**Columns (sheet 3):**

SNPID: ID of lead conditionally independent GWAS variants significantly associated with OA

RSID: RSID of conditionally independent GWAS variants significantly associated with OA

CHR: Chromosome location of conditionally independent GWAS variants significantly associated with OA

BP: base-pair location of conditionally independent GWAS variants significantly associated with OA

C.GENE: Human gene symbol of gene nearest to lead conditionally independent GWAS variants significantly associated with OA

C.DIST: Distance of gene nearest to the lead conditionally independent GWAS variants significantly associated with OA

Human_ensembl_ID: Human Ensembl ID of C.GENE

Mouse_Ensembl_ID: Orthologous mouse Ensembl ID to Human_ensembl_ID

Universe: Gene identified in gene universe as defined in methods section ‘*Enrichment of osteocyte transcriptome signature for orthologs associated with Osteoporosis and OA*’

Osteocyte_signature: Mouse ortholog identified in the *osteocyte transcriptome signature*

LFC: Log2 fold-change in expression data between osteocyte-isolated and whole-bone samples from the *Osteocyte Enrichment* cohort

Gene-level_significant: Genes significantly associated with OA based on gene-wise tests of enrichment (sheet 1)
